# Metformin exerts a synergistic effect with venetoclax by downregulating Mcl-1 protein in acute myeloid leukemia

**DOI:** 10.7150/jca.60208

**Published:** 2021-09-21

**Authors:** Fang-jiao Zhou, Chen-xing Zeng, Wei Kuang, Cong Cheng, Hong-cai Liu, Xue-ying Yan, Xiao-ping Chen, Gan Zhou, Shan Cao

**Affiliations:** 1 Department of Clinical Pharmacology, Xiangya Hospital, Central South University, 87 Xiangya Road, Changsha 410008, PR China.; 2 Institute of Clinical Pharmacology, Central South University, Hunan Key Laboratory of Pharmacogenetics, 110 Xiangya Road, Changsha 410078, PR China.; 3 Engineering Research Center of Applied Technology of Pharmacogenomics, Ministry of Education, 110 Xiangya Road, Changsha 410078, PR China.; 4 National Clinical Research Center for Geriatric Disorders, 87 Xiangya Road, Changsha 410008, Hunan, PR China.; 5 Phase I Clinical Trial Research Center, Xiangya Hospital, Central South University, 87 Xiangya Road, Changsha 410008, Hunan, P.R. China.

**Keywords:** AML, ABT-199, Metformin, Mcl-1, Synergistic anti-tumor effect

## Abstract

**Background:** Recently, one of the specific BH3-mimetics, Venetoclax has been approved by FDA providing new options for newly diagnosed AML patient especially who are unfitted to receive conventional chemotherapy. Though the clinical success of venetoclax has been achieved in clinical outcomes such as complete remission (CR) and overall survival. Acquired resistance to ABT-199 which is induced by the regulation of apoptosis pathway is still an important clinical problem. To this end, the attempt to combine drugs which can reverse the compensatory regulation is urgent.

**Methods:** In three AML cell lines (KG-1, Kasumi-1 and THP-1), the anti-AML effects of the combination of ABT-199 (Venetoclax) and metformin or the two drugs used alone were compared. CCK8 was used to evaluate the cell viability, and flow cytometry was used to estimate the rate of apoptosis, Western blot method was performed to detect apoptosis-related protein levels. In mice experiments, female BALB/c-nu nude mice were subcutaneously injected with THP-1 cells for subcutaneous tumor formation, and the combined effect of ABT-199 and metformin was tested. The evaluation indicators were tumor size, tumor weight, and Ki67 staining. Mouse body weight and HE staining were detected to evaluate liver damage and adverse drug reactions.

**Results:** Both *in vitro* and *in vivo* experiments showed that compared with metformin or ABT-199 alone, the combined use of the two drugs exerts a synergistic effect on promoting apoptosis, thereby producing a strong anti-leukemia effect. Furthermore, after a short incubation time, ABT-199 swiftly increased the expression level of the anti-apoptotic protein Mcl-1, while the combined use of metformin and ABT-199 significantly reduced the level of Mcl-1. Notably, Metformin significantly downregulates the level of Mcl-1 protein by inhibiting its protein production. To less extent, metformin can also downregulate the expression of another anti-apoptotic protein, BCL-xl.

**Conclusion:** Metformin downregulates the expression of anti-apoptotic proteins Mcl-1 and Bcl-xl by inhibiting protein production, and shows a synergistic anti-tumor effect with ABT-199 in acute myeloid leukemia.

## Introduction

Acute myeloid leukemia (AML) is a malignant clonal disease of the blood system, which is characterized by different degrees of differentiation block of myeloid progenitor cells, uncontrolled proliferation of leukemia cells, and inhibition of normal bone marrow hematopoiesis 1. With the clinical application of new molecular targeted drugs, the survival period of AML patients has been extended to a certain extent. However, the shortness of 5-year overall survival rate which is still less than 50% 2-4, along with thefrequent occurrence of relapse or drug resistance 5, 6, challenged the treatment strategy of acute myeloid leukemia (AML). Recently, a new treatment strategy targeting the mitochondrial apoptotic pathway has been applied to elderly or refractory AML patients 7-9. This therapeutic strategy has been adopted by elderly patients who are not appropriate for target therapies or cannot tolerate high-intensity chemotherapeutics, and has become the new treatment trend of AML in recent years.

To maintain their survival, tumor cells often managed to overexpress anti-apoptotic proteins, including Bcl-2, Bcl-xl and myeloid leukemia 1 (Mcl-1) to avoid apoptosis under various stimulation. Due to the specific high expression in hematological malignancies 10, 11, Bcl-2 family protein were targeted to selectively and potently destroy leukemia cells. Many studies reported that Bcl-2 plays a key role in acute myeloid leukemia (AML) cell survival and treatment resistance of conventional chemotherapy 12-14. A highly selective Bcl-2 antagonist, venetoclax, which can competitively abolish the specific binding of pro-apoptotic proteins BIM and Bcl-2 interaction has been developed. This new BH3 mimetics overcomes the shortcomings of low specificity of the previous Bcl-2 antagonists such as ABT-737 and navitoclax 15. Recently, the U.S. FDA approved ABT-199 combined with demethylating agents (decitabine or azacitidine) or low-dose cytarabine to treat newly diagnosed AML patients who cannot tolerate induction chemotherapy or elderly AML patients older than 75 years old 7-9, 16. The complete remission rate of newly-treated AML patients who received ABT-199 combined with HMA treatment was as high as 73%, and the median survival time reached 17.3 months, which showed a clear advantage compared with the 43% complete remission rate and the median survival of 4.5 months of traditional chemotherapy 8. However, although patients will benefit from treatment at first, with the use of drugs, 80% of patients will relapse of drug resistance 8, 17, 18. Therefore, venetoclax still faces a severe problem of resistance.

The mechanisms underlying the sensitivity to Bcl-2 antagonist in AML have not been fully elucidated yet 19. In CLL patients, multiple mutations of Bcl-2 are related to the occurrence of acquired drug resistance, such as Gly101Val, D103Y, Phenol04Ile, Gly33Arg 20, 21, which proved to be unrelated to ABT199 resistant in AML 22. Recently, several groups took advantage of the loss-of-function CRISPR-Cas9 system to screen venetoclax-resistant AML cells and reported that acquired drug resistance is mainly dependent on the compensatory expression of anti-apoptotic proteins Mcl-1 and BCL-xl, which are rapidly elevated after exposure to ABT-199 23-26. Moreover, studies have shown that venetoclax and the Mcl-1 inhibitor S63845 elicit a synergistic effect 22, 27. In light of this, recent studies investigated that various agent synergistically promoted the pro-apoptosis effect of venetoclax by downregulating the expression of Mcl-1 at the transcriptional or post-translational level, such as combined with cyclin-dependent kinase 9 (CDK9) inhibitors (AZD4573), MDM2 inhibitor idasanutlin or with ONC213 analogue imidazine 28-30. Besides, studies also have shown that the expression level of Mcl-1 is of importance in regulating sensitivity of ABT-199. In a report on multiple myeloma (MM), experiments showed that knocking down Mcl-1 can significantly enhance the sensitivity of MM cells to ABT-199 31. Although many studies aim to combine Bcl-2 mimetics with Mcl-1 specific mimetics, it is believed that simultaneously inhibit Bcl-2 and Mcl-1 may bring obvious side effect, since both of them involved in a number of important physiological functions 32, 33. Therefore, there is an urgent need to find a combination plan for reversing the compensatory increase of other anti-apoptotic protein after venetoclax exposure.

Studies have shown that metformin, the first-line hypoglycemic agent, can reduce the risk of many types of cancer and improve overall cancer survival 34-36, and many studies have demonstrated that metformin combined with cytotoxic chemotherapy can target cancer-causing stem cells or drug-resistant cells 37-39. In our previous study, we found that combination of metformin with Cytarabine elicits strong synergistic anti-leukemia effect in AML. Notably, the Bcl-2 family protein expression was decreased after metformin incubation 39, which stimulates us to consider whether metformin plays a role in apoptosis regulation.

The currently recognized anti-tumor mechanism of metformin is AMPK/mTORC1-dependent anti-proliferation and inhibition of mitochondrial complex I 40. By inhibiting mitochondrial complex, metformin can reduce ATP and activity Oxygen (ROS) generation by inhibiting ETC and oxidative phosphorylation, thereby reducing oxidative stress and DNA damage 41. In addition, after inhibiting mitochondrial complex I, metformin can reduce the ratio of NAD+/NADH thus affect the expression of proteins 34, for NAD+ is used as a substrate to participate in the catalytic activities of various enzymes, including histone deacetylases and the regulation of DNA methylase digestive enzyme Tet1, and involved in epigenetic regulation of the expression of a variety of important proteins.

Overall, in the present study, we have developed* in vitro and in vivo* model to test whether metformin and venetoclax, both of which can regulate mitochondrial function, have a synergistic anti-leukemic effect.

## Materials and methods

### Reagents and antibodies

Venetoclax (ABT-199) (1257044-40-8) was purchased from Selleck and Metformin hydrochloride (MB1927-S) was purchased from the Dalian Meilun Biotechnology Co. Cycloheximide was purchased from MCE and MG-132 was obtained from Selleck. Cell Counting Kit (CCK8, B34304) was purchased from Bimake (USA). Annexin V-FITC Apoptosis Detection Kit (C1062M) was purchased from Beyotime (Shang Hai, China). The antibodies β-Actin, BCL-2 and PARP were purchased from Cell Signaling Technology, while BAX and BCL-xl were from ABcone, MCL-1 was purchased from Affinity.

### Cell culture

AML cell lines KG-1, Kasumi-1 and THP-1 were obtained from ATCC (Manassas, VA, USA). KG-1 and Kasumi-1 were cultured in RPMI1640 medium (Gibco, Thermo Fisher Scientific) supplemented with 20% FBS (Cell Max), while THP-1 was cultured with RPMI1640 medium (Gibco, Thermo Fisher Scientific) containing 10% FBS, and they were grown in a humidified atmosphere containing 5% CO_2_ at 37 °C.

### Cell viability assay and Bliss index

Cell proliferation and cytotoxicity were evaluated by the Cell Counting Kit-8 (Bimake) test. KG-1 and Kasumi-1 cells were seeded in 96-well plates at 8×10^4^ per well, while the density of THP-1 was 4×10^4^ per well. Then, after incubating with different concentrations of drugs for 24 hours or 48 hours, CCK8 was added to each 96-well plate and incubated for one hour. Subsequently, the absorbance was detected at 450 nm using a microplate reader (Biotek Instruments Inc.), finally cell viability was calculated. And to evaluate the effect of ABT-199 in combination with metformin, we compared the observed and expected responses obtained from the combination treatment. The Loewe additivity model was used to predict the combined effect of each drug. The expected effect (E_exp_) of the combination was estimated from each separate drug effect:


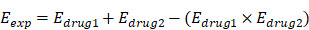


The observed/expected ratio was calculated according to the Bliss method. In this model, the excess above the predicted Bliss index represents the synergistic effect of the combination treatment. The Bliss index was calculated as the observed/expected ratio, where an index of 1, <0 or >1 indicates additive, antagonistic or synergistic effects, respectively 42.

### Apoptosis assay

Apoptosis ratio was assessed using flow cytometric analysis. The cells were collected after drug treatment for 48h, PBS was used to wash treated cells once, and then cells were stained with Annexin V/Propidium Iodide (PI) (Beyotime, Shanghai, China) for 15-20 min. Subsequently, they were detected by the flow cytometer (BD Biosciences, San Jose, CA, USA).

### Quantitative real-time PCR (qPCR)

Trizol reagent (Invitrogen, Carlsbad, CA, USA) was used to extract total RNA from cell lines, cDNA synthesis was performed using the Reverse Transcription System (Takara, Japan). TB Green qPCR Mix (Takara, Japan) was used to perform real-time PCR. Reactions were performed on a LightCycler®480 II/96 (Roche Diagnostics Ltd, Rotkreuz, Switzerland). The relative expression of target genes was normalized to β-Actin and calculated using the 2^-ΔΔCt^ method. All real-time PCR reactions were performed in triplicate. The primer sequence was as follows:

- Human-β-actin-F 5′ CATGTACGTTGCTATCCAGGC 3′,
- Human-β-actin-R 5′ CTCCTTAATGTCACGCACGAT 3′,
- Human-BCL-2-F 5′ GGTGGGGTCATGTGTGTGG 3′,
- Human-BCL-2-R 5′ CGGTTCAGGTACTCAGTCATCC 3′,
- Human-MCL-1-F 5′ GTGCCTTTGTGGCTAAACACT 3′; and,
- Human-MCL-1-R 5′ AGTCCCGTTTTGTCCTTACGA 3′.

### Western blotting

The treated cells were collected by centrifugation at 800 rpm, and then washed three times with PBS at 1000 rpm. All the proteins were extracted from cultured cells by RIPA (Beyotime, Shanghai, China), using a protease inhibitor and phosphorylase inhibitor cocktail (Bimake, Shanghai, China). The protein concentration was measured via BCA Protein Assay Kit (Pierce, Rockford, IL, USA).

Total cell lysate per sample was loaded on a sodium dodecyl sulfate-polyacrylamide gel electrophoresis (SDS-PAGE) gel and separated by electrophoresis. The western blot was blotted onto 0.2 uM PVDF membrane using a wet transfer device (Bio-Rad). Block the blot in a 5% milk powder solution diluted in TBST (Tris buffered saline/0.05% Tween 20), incubated with indicated antibody, and signal detected with a horseradish peroxidase (HRP)-conjugated secondary antibody and enhanced chemiluminescence (ECL) detection (Bio-Rad).

### Nude mouse xenograft model

The animals of our study were approved by the Institutional Animal Care and Use Committee of the Central South University School of Medicine (No. 2020sydw0984). Female nude mice (five weeks old; average weight, 16 g) were obtained from Experimental Animal Laboratories (Changsha, China). THP-1 cells were resuspended in PBS and Matrigel (5×10^6^cells/mice), and then injected into the right side of the foreleg of each mouse subcutaneously at an inoculation depth of 1 cm. After tumor formation, the mice were divided into four different groups (vehicle, ABT-199 (100 mg/kg), metformin (200 mg/kg), ABT-199(100 mg/kg)/metformin (200 mg/kg)). About 9 days later, dosing experiment after the tumor growth to about 50 mm^3^ (vehicle group used saline and CMC-Na to replace, ABT-199 was made into a suspension with 0.5% CMC-Na). About 14 days after administration, all mice were sacrificed by cervical dislocation, tumors were dissected and photographed. Tumor sizes in mice were measured every 2 days using a digital caliper, and all tumor volume were calculated using the following formula: V = length × (width)^2^/2.

### Statistical analysis

All the experiments were performed in triplicates. All data were reported as the mean ± standard error of the mean (SEM). Statistical analysis was performed using the student's t-test and the one-way ANOVA test. *P* value < 0.05 was considered statistically significant.

## Results

### Venetoclax and metformin elicit a synergistic growth inhibition effect in AML cells

**Figure 1:**
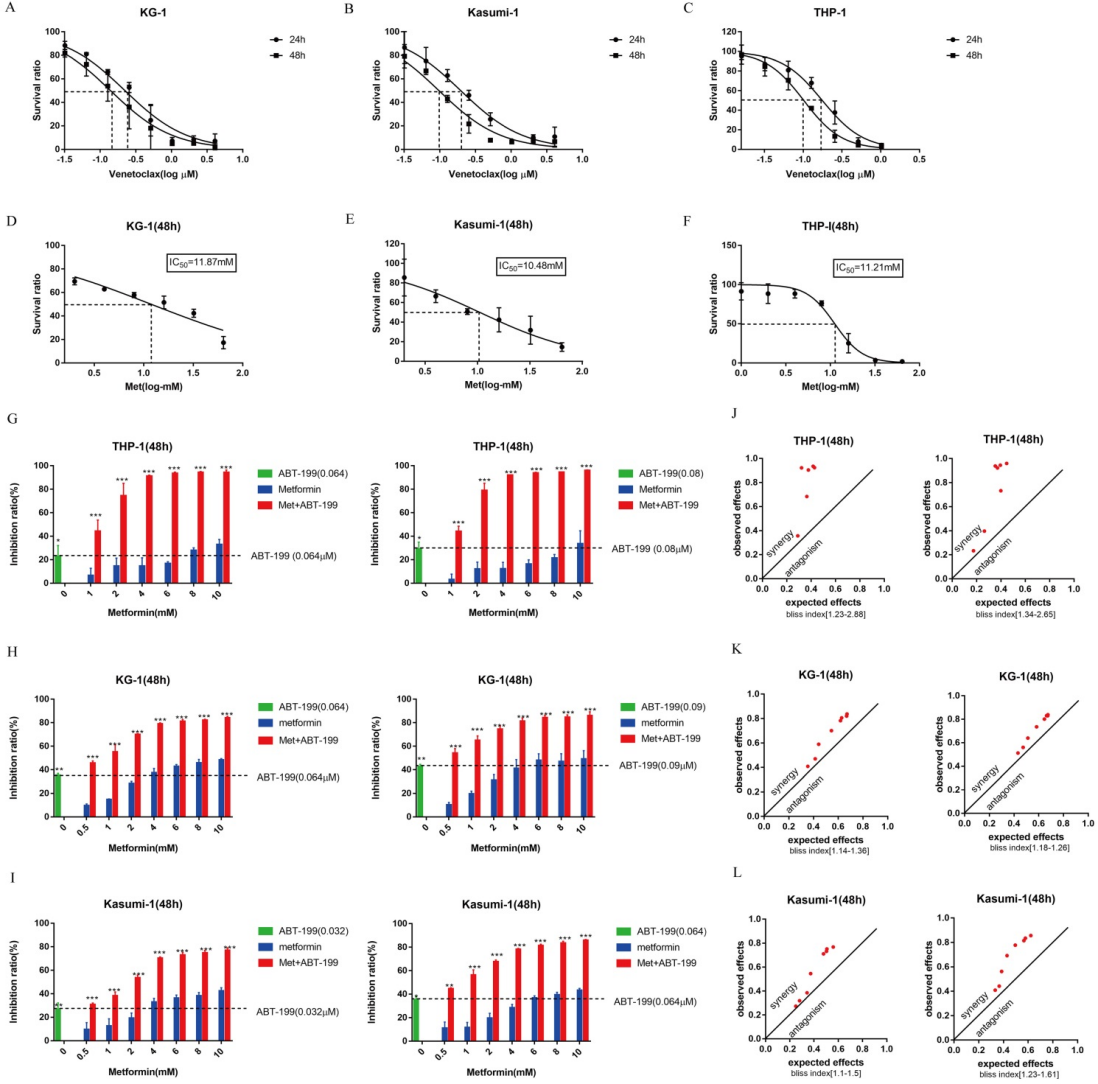
** Both ABT-199 and metformin inhibited growth of AML cells and the combined treatment has a synergetic effect. (A-C)** The IC_50_ of ABT-199 in KG-1 (A), Kasumi-1 (B) and THP-1 cells (C) for 24, 48 hours. **(D-F)** The IC_50_ of metformin in KG-1 (D), Kasumi-1 (E) and THP-1 cells (F) for 48 hours. **(G)** The inhibition ratio of ABT-199, metformin and their combination in THP-1 cells when the concentration of ABT-199 was fixed at 0.064 µM (left) and 0.08 µM (right). **(H)** The concentration of ABT-199 was fixed at 0.064 µM (left) and 0.09 µM (right), the inhibition ratio of different concentrations of metformin and the combination of the two drugs in KG-1. **(I)** The inhibition ratio of ABT-199, metformin and the combination in Kasumi-1 cells when the concentration of ABT-199 was fixed at 0.032 μM(left) and 0.064 µM (right) (*P < 0.05; ** *P* < 0.01; *** *P* < 0.001). **(J)** The bliss index of ABT-199 fixed at 0.064 µM (left) and 0.08 µM (right) in THP-1. **(K)** The bliss index of ABT-199 fixed at 0.064 µM (left) and 0.09 µM (right) in KG-1. **(L)** The bliss index of of ABT-199 fixed at 0.032 µM (left) and 0.064 µM (right) in Kasumi-1.

First, the CCK8 assays were performed to identify the IC_50_ values of ABT-199 for 24h and 48h in KG-1, Kasumi-1 and THP-1 cell lines. The IC_50_ values of ABT-199 for 24h and 48h were 0.223 μM and 0.140 μM in KG-1 cells, 0.197 μM and 0.097 μM in Kasumi-1 cells, and 0.174 μM and 0.153 μM in THP-1 cells (Figure 1A-C). Concentrations around IC_40_ of ABT-199 were used in different cell lines in the subsequent studies. After 48h incubation, the IC_50_ values of metformin were 10.48 mM in Kasumi-1, 11.87 mM in KG-1and 11.21 Mm in THP-1, respectively (Figure 1D-F).

To determine whether metformin could enhance the growth suppression effect of ABT-199, we analyzed the cell viability in these three AML cells. Various concentrations of metformin enhanced the growth inhibition effect of ABT-199 after 48h incubation in all AML cell lines (Figure 1G-I). The bliss index which larger than 1 can be used as an index of synergy was calculated. The results indicated that the combination of ABT-199 and metformin has a potent synergistic effect in the three AML cells (Figure 1J-L). The Bliss index in all three cells is larger than 1 when the two drugs are used in combination, even when the concentration of metformin is relatively small (such as 1 mM and 2 mM). Moreover, the Bliss index in THP-1 was the largest, suggesting that the best synergistic effect presented in THP-1 cell.

### Combination of metformin and ABT-199 induce a synergistic pro-apoptotic effect on AML cells

**Figure 2:**
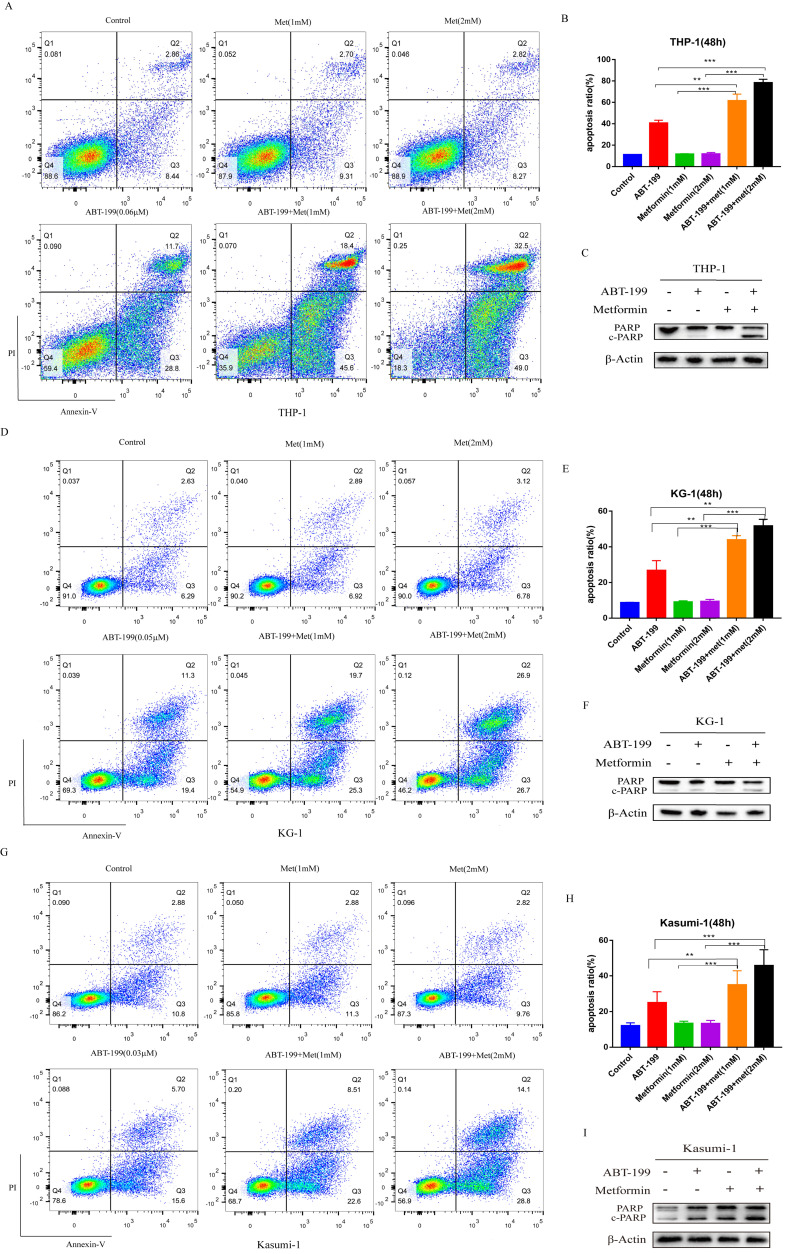
** ABT-199 and metformin co-treatment has a synergistic pro-apoptosis effect on AML cells.** THP-1 (A), KG-1 (D) and Kasumi-1 (G) were treated with ABT-199, metformin, or ABT-199/metformin for 48 hours, the fraction of apoptotic cells was analyzed by Annexin V/PI staining and flow cytometric analysis. The statistical result of the (A), (D) and (G) graph results were shown in (B), (E) and (H) (*P < 0.05; ** P < 0.01; *** P < 0.001). The apoptosis protein PARP (c-PARP) was examined by western blot in THP-1 (C), KG-1 (F) and Kasumi-1 (I).

Next, we determined whether metformin can promote the effect of ABT-199 in AML cells by inducing apoptosis. The apoptosis level of AML cells was detected by flow cytometry. Concentrations were used around IC_40_ of ABT-199 in different cell lines (0.06 µM in THP-1, 0.05 µM in KG-1, and 0.03 µM in Kasumi-1). Notably, the low concentrations of Metformin (1 mM and 2 mM) which only induce negligible apoptosis effect were used in all cell lines. In THP-1 cells (Figure 2A, B), compared with ABT-199 alone which induced the 40.59% apoptosis, the percentage of apoptotic cells reached to 61.33% (*P*<0.01) after combining with 1mM metformin and reached to 78.27% (*P*<0.01) after combining with 2 mM metformin. In the other two cell lines, the combination of metformin and ABT-199 also caused a significantly higher apoptosis ratio than ABT-199 alone (Figure 2D, E, G, H), similarly, metformin alone did not cause obvious apoptosis in these two cell lines.

The expression of apoptosis related protein c-PARP (cleaved-PARP) was detected by western blot to confirm the apoptosis effect. Results indicated that when metformin (2 mM) cooperated with ABT-199, the expression of c-PARP protein in KG-1 and Kasumi-1 cells was significantly increased than that induced by ABT-199 alone (Figure 2F, I). Moreover, in THP-1 cells, the expression of c-PARP protein increased more obviously after the combination of the two drugs (Figure 2C), which was consistent with the results of flow cytometry. All above results suggested that ABT-199/metformin co-treatment exerts synergistic pro-apoptotic effects on AML cells.

### Metformin reduced the protein expression of Mcl-1 in synergy with ABT-199 instead of reducing ATP production

**Figure 3:**
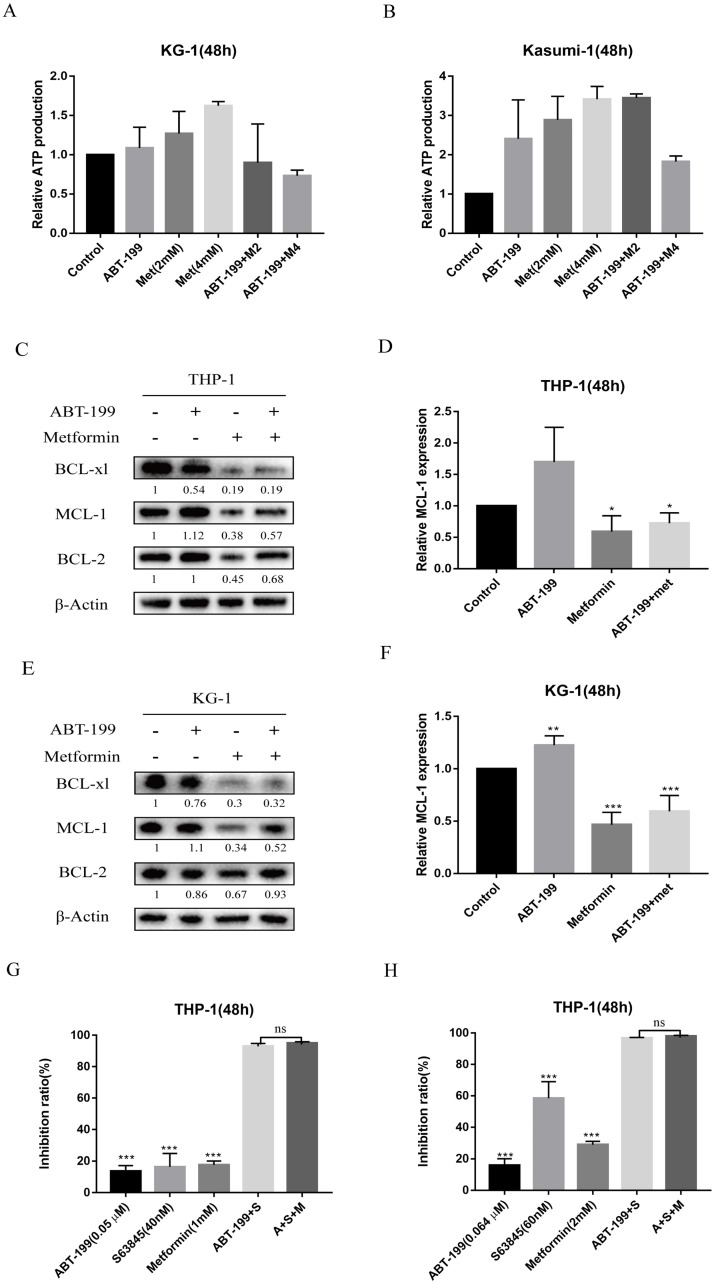
** The synergistic pro-apoptotic effect of the combination of ABT-199 and metformin is achieved by reducing ABT-199 induced compensatory increase of Mcl-1.** The ATP production was measured with ATP Assay Kit by Biotek Synergy LX after 48 hours treatment of ABT-199 and metformin in KG-1 (A) and Kasumi-1 (B). The level of Mcl-1, Bcl-2, BCL-xl proteins were detected using western blot after ABT-199, metformin, ABT-199/metformin treated for 48 hours in THP-1 (C) and KG-1 cells (E). The statistical result of Mcl-1 in the (C) and (E) graph results was shown in (D) and (F). (*P < 0.05; ** *P* < 0.01; *** *P* < 0.001). THP-1 cells were treated with ABT-199, S63845 and metformin alone or in combination, the cell viability was measured and the inhibition rate was calculated. (* *P* < 0.05; ** *P* < 0.01; *** *P* < 0.001).

As many studies have reported that the regulation of energy metabolism in cancer cells by metformin is one of the most acknowledged anti-cancer mechanisms underlying metformin. Thus, the production of ATP after treated with the two drugs was measured by ATP Assay Kit (Biotek Synergy LX). The results as shown in Figure 3A and 3B, indicated that after 48 hours treatment of metformin monotherapy, the ATP production of AML cells did not decrease, and the combination with ABT-199 did not decrease ATP production compared with the control group. This result indicated that energy metabolism, indicated by ATP level, may not be the mechanism underlying the synergistic effect of metformin with ABT-199.

Next, we detected the expression level of three anti-apoptotic proteins Bcl-2, Mcl-1 and BCL-xl, because the up-regulation of Mcl-1 and BCL-xl are the most crucial factors leading to ABT-199 resistance. As shown in Figure 3C and E, the expression of Mcl-1 protein in AML cells increased when ABT-199 was treated alone, and metformin alone could significantly reduce the expression of Mcl-1, and when the two drugs were used in combination, the expression level of Mcl-1 was significantly lower than that of ABT-199 alone (Figure 3D, F). ABT-199/metformin co-treatment also reduced the BCL-xl protein expression, but did not change the expression level of Bcl-2 protein (Figure 3C, E). The above results indicated that ABT-199 can cause a compensatory increase in Mcl-1 protein in AML cells, and metformin can reduce the expression level of Mcl-1 induced by ABT-199, thereby synergistically exerting a better therapeutic effect.

Further, to determine whether the synergy between metformin and ABT-199 is achieved by targeting Mcl-1, THP-1 cells were treated with S63845, a selective inhibitor of Mcl-1, in combination with ABT-199 with or without metformin, and the inhibition ratio were examined. The results showed that different concentrations of ABT-199 and S63845 presented a strong synergistic suppression in THP-1 cells, but the effect did not further enhance after the addition of metformin (Figure 3G, H), which in part indicated that the synergistic effect of metformin and ABT-199 is based on the inhibition of Mcl-1.

### Metformin reduced the expression of Mcl-1 in AML cells by inhibiting protein production

**Figure 4:**
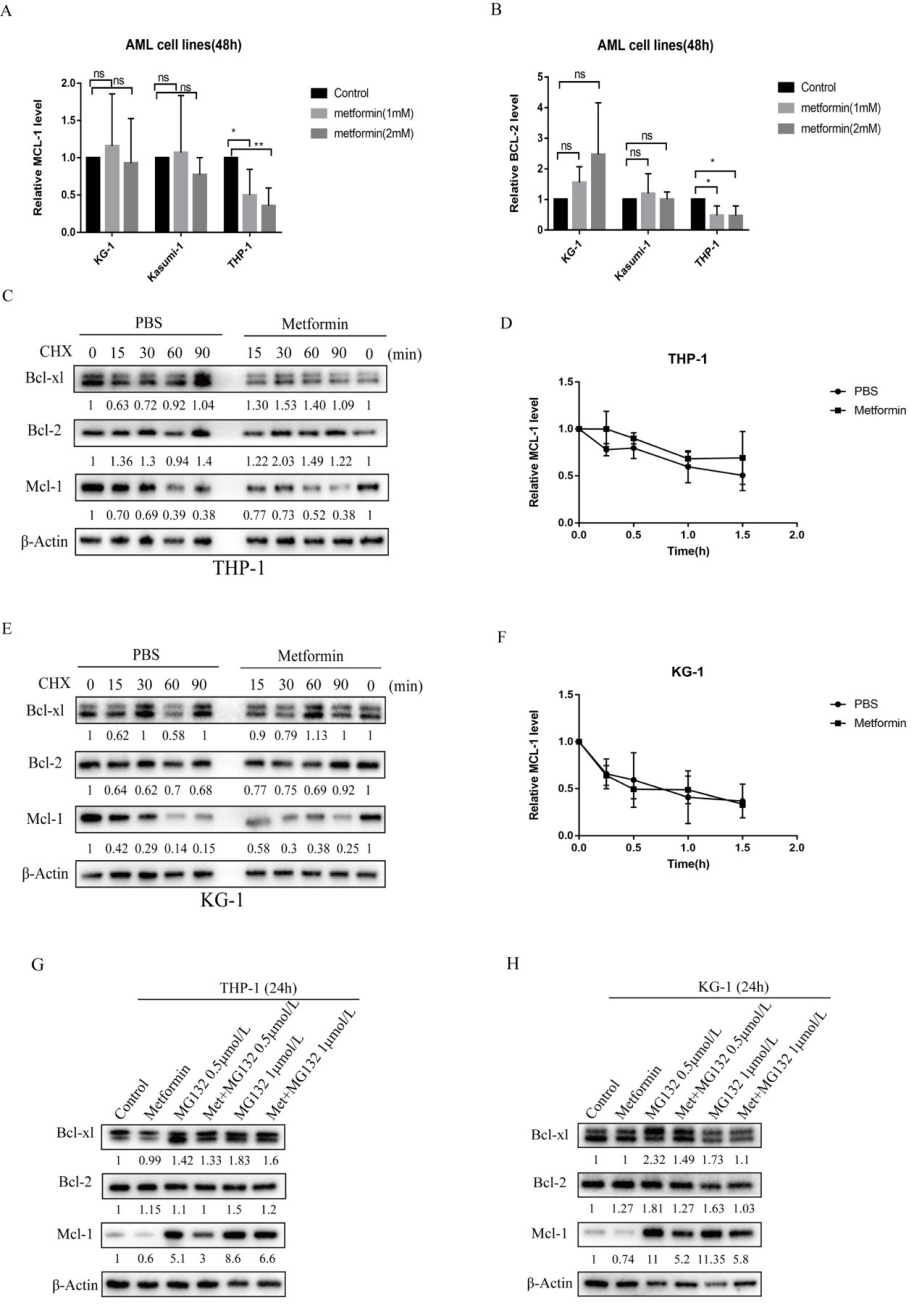
** Metformin reduced the expression of Mcl-1 in KG-1 and THP-1 cells by inhibiting protein production, and also decreased the transcription level of Mcl-1 in THP-1.** The transcription level of Mcl-1 (A) and Bcl-2 (B) were measured after treatment with metformin for 48 hours in KG-1, Kasumi-1 and THP-1 cells. The level of Mcl-1, Bcl-2, Bcl-xl proteins were detected by western blot after cycloheximide, metformin, cycloheximide/metformin treatment for 48 hours in THP-1 (C) and KG-1 (E). The time-course degradation of Mcl-1 protein in the figure (C) and figure (E) graph were plotted in (D) and (F). The results are shown as bar graphs with the mean± s.d.,n =3 independent experiments. The statistical significance was determined by Student's t-test. The level of Mcl-1, Bcl-2, Bcl-xl proteins were detected by western blot after metformin, MG-132, metformin/MG-132 treatment for 48 hours in THP-1 (G) and KG-1 (H).

To elucidate the molecular mechanism by which metformin reduces the level of anti-apoptotic proteins, we first measured the transcription level of Mcl-1 and Bcl-2 after metformin treatment. Following metformin treatment, Mcl-1 transcript levels remained unchanged in KG-1 and Kasumi-1 cells (Figure 4A), suggesting that transcriptional regulation of Mcl-1 did not play a prominent role in KG-1 and Kasumi-1. However, the transcription level of Mcl-1 decreased in THP-1 cells after treated with metformin, and the transcription level of Bcl-2 also decreased slightly (Figure 4A, B). Then we treated KG-1 and THP-1 cells with the protein translation inhibitor cycloheximide (CHX) for up to 90 minutes in the absence or presence of metformin to see if metformin can promote protein degradation in AML cells. The results showed that there is little difference in the degradation rate of Mcl-1 protein with or without metformin treatment in THP-1 and KG-1 cells, and the same phenomenon was observed in Bcl-xl and Bcl-2 proteins (Figure 4C-F), which indicates that metformin reduces the expression of anti-apoptotic proteins such as Mcl-1 not by promoting degradation. Furthermore, to determine whether metformin affected the ubiquitin-proteasome pathway, THP-1 and KG-1 cells were treated with the proteasome inhibitor MG-132 for 24 hours with or without metformin. Mcl-1 protein level was increased after incubation with 0.5 mmol/L and 1 mmol/L MG-132 for 24 hours, whereas, downregulation of Mcl-1 protein was detected after addition of metformin. This phenomenon was also observed in Bcl-2 and BCL-xl protein to a less extent (Figure 4G, H). These results demonstrate that metformin reduces the expression of anti-apoptotic proteins such as Mcl-1 via inhibiting the protein production.

### Synergistically effect of ABT-199 and metformin *in vivo*

**Figure 5:**
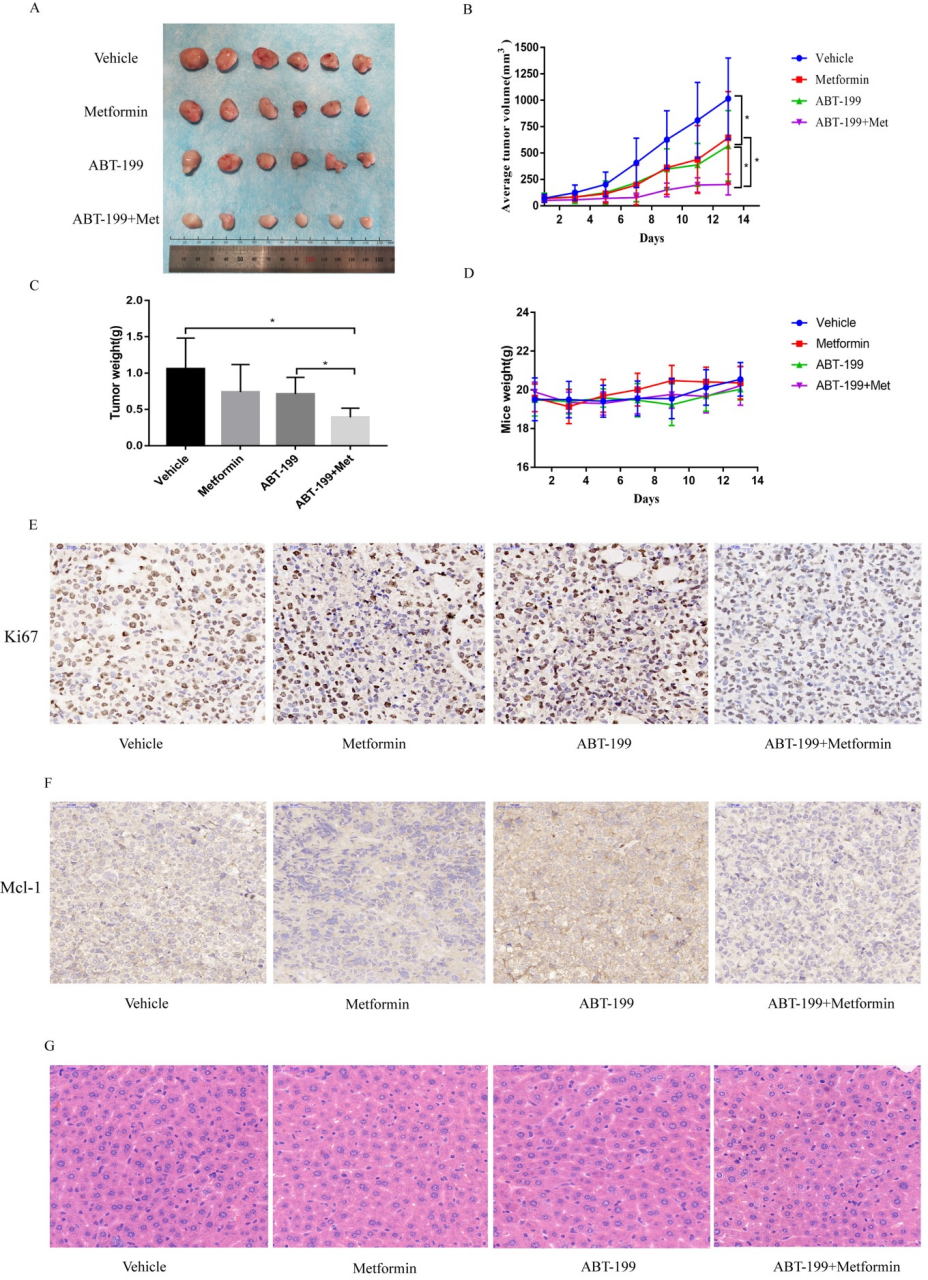
** ABT-199 and metformin co-treatment effectively repressed tumors in nude mouse xenograft model.** Nude mice injected with THP-1 cells subcutaneously were treated with ABT-199 (100 mg/kg/d) by mouth (P.O.), metformin (200 mg/kg/d) intraperitoneally injected (either alone or in combination) or vehicle control when the tumor volume reached about 50 mm^3^. **(A)** Images of the tumors of four treatment groups recorded at day 14. **(B)** The average tumor volume (mm^3^) curve of the four groups measured every 2 days. **(C)** The tumor weight curve in nude mice of the four treatment groups (* *P* < 0.05; ** *P* < 0.01; *** *P* < 0.001). **(D)** The toxic side effect of drugs on nude mice was assessed by the change in body weight in mice. **(E)** Immunohistochemistry and Ki67 antibody were used to measure the proliferation of AML cells in nude mouse xenograft model after CMC-Na, ABT-199, Metformin, ABT-199/Metformin treated. **(F)** Immunohistochemistry and Mcl-1 antibody were used to measure the protein level of Mcl-1 in **(G)** nude mouse xenograft model with four different treatments. **(H)** Liver tissue of the four groups was H&E stained to detect liver damage after drug treatment.

To explore the anti-leukemia effect of the combination of ABT-199 and metformin *in vivo*, BALB/c-nude mice were subcutaneously inoculated with THP-1 cells and then drug treatment was given when the tumors grew to about 50 mm^3^. All mice were randomly divided into 4 groups with different treatment (n=6), this experiment was terminated after 14 days of drug treatment. The average size of tumor in vehicle group was 1015.19 ± 385.03 mm^3^ which was significantly larger than the other administration groups. The average tumor volume of the ABT-199 and metformin monotherapy groups were 568.1 ± 333.97 mm^3^ and 644.9± 438.10 mm^3^, respectively. While the average size of tumor in ABT-199/metformin co-treatment group was significantly reduced to 201.8 ± 99.08 mm^3^ (Figure 5A, B) (*P*<0.05). Meanwhile, the average tumor weights of the four groups were also measured. Like the tumor volume, the tumor weights of the ABT-199 and metformin single-agent groups were lower than the vehicle group, while the combined drug group was the lightest weight among four groups (Figure 5C). These results demonstrated that compared with ABT-199 alone, the combined treatment of metformin and ABT-199 elicits a better anti-leukemia effect. The anti-proliferation effect was evaluated by immunohistochemistry result from ki67 (Figure 5E) dyeing. As showed in Figure 5E that two drugs alone have little effect on the proliferation of AML cells compared with the vehicle group, whereas, the ABT-199 and metformin co-treatment group presented an obvious decrease of proliferation cells.

Besides, the Mcl-1 level was evaluated by the immunohistochemistry to identify the mechanism of the combination. The results (Figure 5F) indicated that the expression level of Mcl-1 in ABT-199 group was increased compared with the vehicle group, and downregulation was detected in the metformin group and the combination drug group, which is in line with the protein results *in vitro*.

Finally, we detected the side effects of this combination. The total weight of mice was measured in the four groups every two days, and the results showed no difference in these four groups, and no weight loss was found in any group (Figure 5D). HE staining was used to assess the degree of liver damage of drugs in mice, and the result indicated that liver tissue of mice in ABT-199 group, metformin group and ABT-199/metformin group had no significant toxic side effect compared with mice in vehicle group (Figure 5G), which suggested ABT-199 and metformin treatment did not cause liver damage.

## Discussion

Targeting Bcl-2 proved to be a major advance in the treatment of AML. At present, Venetoclax, a selective Bcl-2 antagonist, has been approved for the treatment of elderly AML, and its efficacy is better than traditional chemotherapy drugs 9. However, Venetoclax is prone to acquired resistance after long-term treatment. Most studies report that the main reason for its resistance is the compensatory increase of Mcl-1 43, which was verified in our results. Meanwhile, many new BH3 mimetics of the Bcl-2 family have emerged in recent years, such as selective Mcl-1 antagonists, which have been observed having good curative effects in treatment of AML 44, but there is also the problem of acquired resistance.

Many studies aim to combine Bcl-2 mimetics with Mcl-1 specific mimetics, Mcl-1 is another anti-apoptosis protein which is believed to be involved in a number of important physiological functions. Although the attempt of combined ABT-199 with Mcl-1 inhibitors have achieved success to certain extent, there is extensive suspicions about having obvious adverse reactions 45. We found metformin can decrease the compensatory increase of Mcl-1 and BCL-xl to less extent when combined with ABT-199 and without causing obverse side effects. Our exploration found that the combined mechanism of these two drugs in AML cells is different, metformin reduced the expression of Mcl-1 in both KG-1 and THP-1 cells not by promoting protein degradation but by inhibiting protein synthesis. But it decreased the transcription level of Mcl-1 in THP-1 not in KG-1 cells. This phenomenon may explain our previous experiments that the combined effect of metformin and ABT-199 in THP-1 cells is better than that in KG-1 and Kasumi-1 cells.

ABT-199 can trigger cell apoptosis by regulating mitochondrial function. Interestingly, metformin functioned as inhibitors of mitochondrial electron transport chain to elicit the anti-tumor effect. Both of their function converged in mitochondria, which is involved in a variety of important physiological functions, such as the generation of energy substance ATP, reactive oxygen species (ROS) production, biomacromolecule synthesis, signal transduction, and apoptosis 46. Inconsistent with the study on the combination of metformin, venetoclax and cytarabine 47, our results demonstrated that the synergistic effect of the two drugs in promoting apoptosis is unrelated to the level of ATP, but by decreasing the expression level of apoptotic protein Mcl-1. Recently, several studies have confirmed that metformin reduce the level of NAD+, which may participate in the catalytic activities of various enzymes, including histone deacetylases and the regulation of DNA methylase digestive enzyme Tet1. Thus, the further exploration of whether metformin increased the expression of Mcl-1 is on the basis of regulating of epigenetic activity is needed.

As so far, there are few reports on the combination use of ABT-199 and metformin in AML, an article in 2016 stated that biguanide drugs can promote the apoptosis of ABT-737 on leukemia cells through the mitochondrial electron transport chain 48. Different from our research, this article uses ABT-737 instead of Bcl-2 selective antagonist ABT-199. Their entire experiment is not aimed at AML, and the mechanism of metformin we found is not consistent with their research. There is also an article on AML that mentioned the two drugs, but the combination of them is less involved and the research is mainly focus on the perspective of metabolism 49.

In conclusion, we verified the combined effect of ABT199 and metformin *in vitro and in vivo* model, and found that metformin can promote the pro-apoptotic effect of ABT-199 by regulating apoptosis-related proteins, but the underlying mechanisms warrant further investigation. These preliminary results provided new insights for the treatment of AML patients, but still has several limitations. First, although the concentration of metformin in the present study is much lower than that in solid tumors studies, it is still a relatively high concentration in clinical use. Secondly, the combination of ABT-199 and metformin should be verified in more clinically relevant model, such as patient-derived tumor xenograft model. Thirdly, the underlying mechanism of ABT-199/metformin synergy needs further exploration.
